# Autologous Blood Versus Talc Pleurodesis and the Influence of Non-steroidal Anti-inflammatory Drugs

**DOI:** 10.1093/icvts/ivaf264

**Published:** 2025-10-31

**Authors:** Finn Amundsen Dittberner, Giuliana Moreano Diaz, Lars Svend Börnsen, Peter Bjørn Licht

**Affiliations:** Department of Cardiothoracic Surgery, Odense University Hospital, Odense 5000, Denmark; Department of Clinical Pathology, Odense University Hospital, Odense 5000, Denmark; Department of Clinical Pathology, Odense University Hospital, Odense 5000, Denmark; Department of Cardiothoracic Surgery, Odense University Hospital, Odense 5000, Denmark

**Keywords:** pleurodesis, autologous blood, talc, non-steroidal anti-inflammatory drugs

## Abstract

**Objectives:**

To compare the extent of pleural inflammation and fibrosis induced by autologous blood vs talc pleurodesis in an exploratory experimental model and evaluate effects of postoperative non-steroidal anti-inflammatory analgesics on pleurodesis formation.

**Methods:**

Twenty-eight Sprague Dawley rats underwent intrapleural instillation of autologous blood on one side and talc on the contralateral side. They were sacrificed at 2, 4, 6, 15, or 30 days for macroscopic and histopathological analysis. Eight animals in the late euthanasia groups received oral Ibuprofen postoperatively. A pathologist, who was blinded to the interventions assessed all animals for macroscopic adhesions in the chest as well as microscopic evaluation for inflammation and fibrosis.

**Results:**

We found no significant differences between autologous blood and talc regarding macroscopic adhesion scores, or grading of inflammation and fibrosis. The inflammatory response peaked earlier after autologous blood compared with talc. Fibrosis progressively increased after both interventions. Ibuprofen reduced inflammation and fibrosis in both types of pleurodesis. Statistically significant reductions in fibrosis were seen after 15 days in the talc group (*P* = .008) and after 30 days in the autologous blood group (*P* = .024).

**Conclusions:**

Autologous blood and talc pleurodesis induce comparable inflammatory responses and fibrosis in this experimental model suggesting that the mechanism of autologous blood patch for prolonged air leakage is not just a mechanical plug effect. Ibuprofen reduced all inflammatory responses after both interventions suggesting that non-steroidal anti-inflammatory drugs may impair pleurodesis formation.

## Graphical abstract

**Figure:**
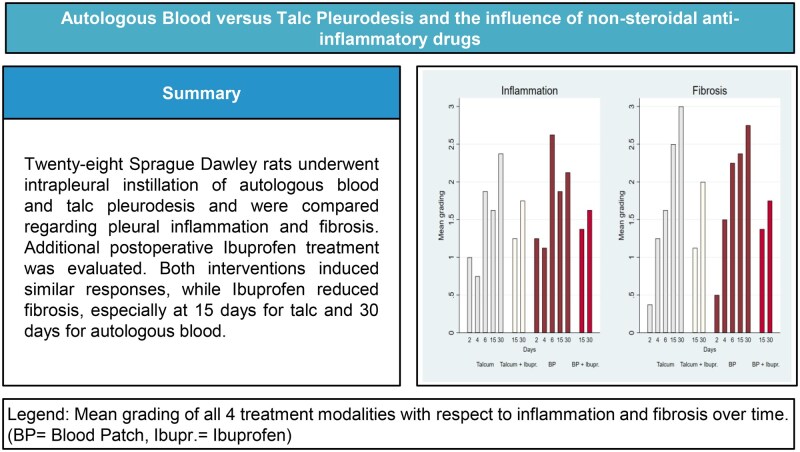

## INTRODUCTION

Persistent air leakage is a common clinical challenge in patients with pneumothorax secondary to pulmonary disease or after thoracic surgery.^1^ Autologous blood pleurodesis or blood patch may be used to shorten the duration of air leakage although its mechanism remains unclear. It may act by forming a mechanical plug over the pleural defect,^2^^,^^3^ supported by rapid air leak resolution in some situations. Animal studies, however, suggest that it may also induce adhesions, inflammation, and fibrosis,^4^^,^^5^ indicating that its mechanism may extend beyond simple mechanical sealing.

Various modes of pleurodesis have been investigated after lung resections with prolonged air leakage^6–13^ as well as a potential method to prevent recurrent spontaneous pneumothorax^12–19^ with reported success rates of 60%-90%.^6^^,^  ^15^ Talc remains the most commonly used agent in both Europe and the United States,^19^ but the search for an optimal pleurodesis agent remains ongoing. Effective pleurodesis is believed to depend on fibrin adhesion and subsequent fibrosis to create a lasting bond between the pleural membranes, with inflammation as a key driver although multiple pathways may be involved.^20^^,^^21^ Despite its efficacy, chemical pleurodesis often causes adverse effects such as pain, dyspnoea, fever, granulomatous deposits, and impaired lung function, both when applied in prolonged air leakage^9^ and spontaneous pneumothorax.^17^ This has prompted growing interest in search for safer and equally effective alternatives.

Furthermore, it is still controversial whether non-steroidal anti-inflammatory drugs (NSAIDs) interfere with pleurodesis. NSAIDs are very useful analgesics post-surgery but inhibit platelet cyclooxygenase and block thromboxane A2 formation, which is important for clot formation. Theoretically, this blocking may therefore impair the sealing effect of blood pleurodesis and reduce the inflammatory response needed for adhesion formation after both autologous blood and chemical pleurodesis.^5^ Experimental and clinical studies have demonstrated reduced pleurodesis efficacy with NSAID use.^5^^,^  ^22^

Given the side effects of chemical agents, alternative approaches are needed. Autologous blood pleurodesis appears to be promising, especially for recurrent pneumothorax or prolonged postoperative air leakage.^10^^,^^11^^,^  ^13^^,^  ^18^ To our knowledge, no previous animal study has examined the early histopathological response to blood pleurodesis with the earliest such investigation reported in the literature assessed changes at 7 and 21 days,^5^ while the remaining previous studies^2–4^ evaluated histopathological effects only at day 30. In an exploratory setting, the present study aimed to evaluate and compare the extent of early pleural inflammation and fibrosis induced by autologous blood and talc slurry as well as late changes, both with and without the administration of ibuprofen to elucidate potential differences in their mechanisms of action and inflammatory profiles.

## METHODS

The Animal Experiments Inspectorate under the Ministry of Food, Agriculture and Fisheries of Denmark approved this study (permission number: 2022-15-0201-01289), and we conducted all procedures in compliance with national and institutional guidelines for animal care and welfare. We did all experiments at the Biomedical Laboratory of Odense University Hospital and the University of Southern Denmark between January 18 and February 16, 2023. We used 28 Sprague Dawley rats (median weight 600 g, range 570-680 g) for this study and divided them into 5 experimental groups based on the timing of sacrifice. All animals underwent unilateral intrapleural administration of venous autologous blood (1.5 mL/kg). We used the contralateral hemithorax as positive control with intrapleural injection of sterile talc slurry (Imerys Talc Italy S.p.A) (70 mg/kg in 1 mL/kg saline). We sacrificed groups 1 to 5 (each containing 4 animals) on day 2, 4, 6, 15, and 30, respectively. We expanded groups 4 and 5 with 4 additional animals who received oral ibuprofen postoperatively (group 4a and 5a, respectfully) to assess the potential inhibitory effect of NSAIDs on pleural inflammation and fibrosis formation by comparing findings in subgroups 4 and 5 (without NSAIDs) with 4b and 5b (with NSAIDs).

### Technical aspects

We induced anaesthesia via subcutaneous injection of a mixture of fentanyl (236 mL/kg), fluanisone (7.5 mg/kg), and midazolam (3.75 mg/kg), administered at 0.3 mL/100g. To prevent hypothermia, we placed the animals on a heating pad. We used subcutaneous administration of 3 mL saline in the neck fold for hydration, and we applied neutral eye ointment to prevent corneal dehydration. The chest was shaved, and the designated intercostal space was disinfected with 70% alcohol. The animal was positioned in either left or right lateral decubitus and local anaesthesia with Bupivacaine (0.3% 1 mL/kg) was administered subcutaneously over a 5-10mm^2^ area at the designated intercostal space.

**Figure 1.**
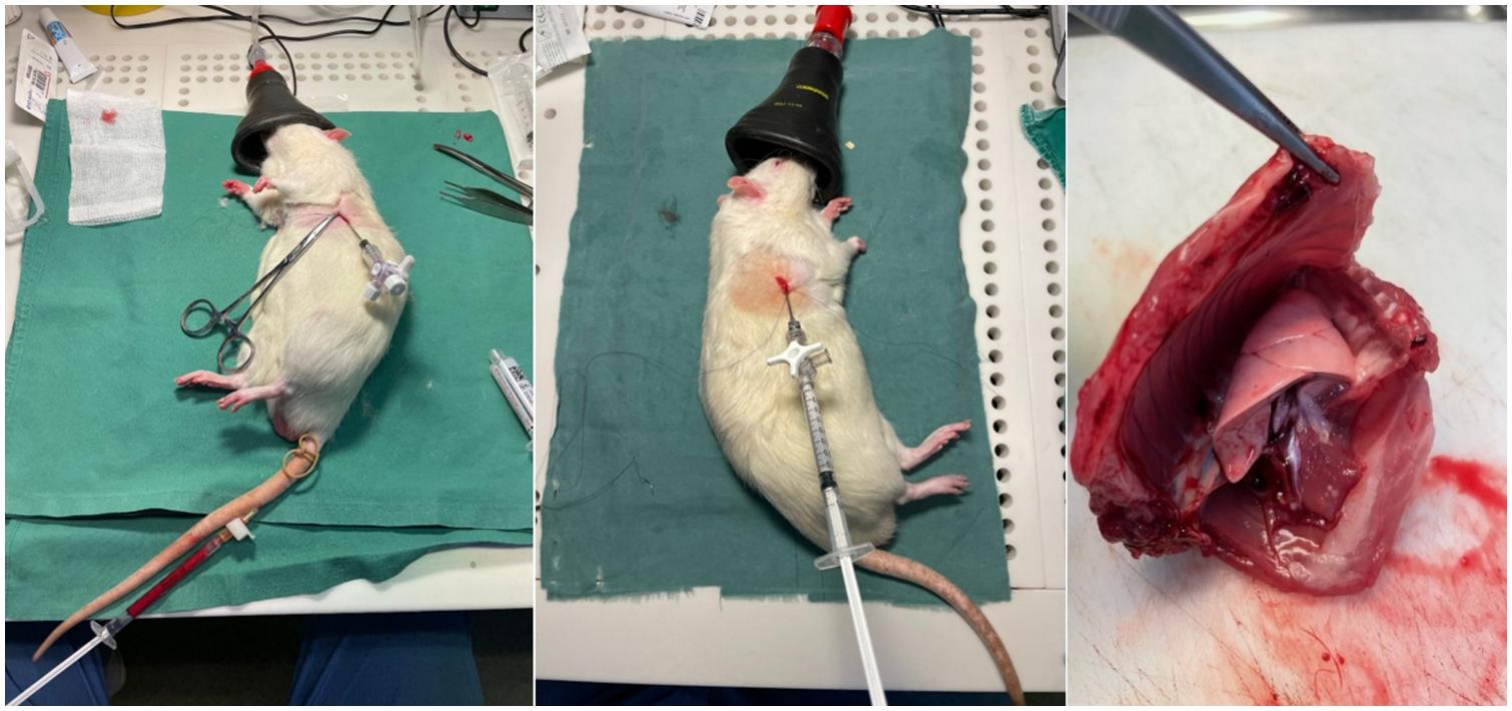
Lateral tail vein puncture with corresponding autologous blood pleurodesis (left). Talc instillation (middle). The thoracic cavity removed en bloc with corresponding macroscopic examination (right)

We then made a 2-3 mm incision and introduced a blunt-ended stainless steel oral gavage feeding tube, sealed with a 3-way stopcock, into the pleural cavity and held it in place by a simple purse-string suture. The lateral tail vein was then located and punctured with a 23-gauge butterfly needle to collect 1.5 mL/kg of venous blood. We immediately instilled the collected blood into the pleural space via the chest catheter (**Figure 1**). Air was exsufflated through the stopcock until vacuum, after which the chest tube was removed, and the incision was closed with the purse-string suture.

The animals were repositioned into the contralateral lateral decubitus position, and the same chest tube application procedure was repeated with sterile talc slurry (Imerys Talc Italy S.p.A) 70 mg/kg in 1 mL/kg saline instead of blood before chest tube removal and wound closure (**Figure 1**). All animals then underwent a standardized rotation protocol to ensure even distribution of autologous blood and talc slurry across the pleural surfaces before returning to their cages.

We used oral buprenorphine (0.2 mg) for postoperative analgesia. We mixed the analgesic into 1 g of Nutella, and if the animal refused oral intake, we injected subcutaneous buprenorphine (0.05 mg/kg) every 6 hours for 48 hours. Animals in the NSAID group received additional analgesia with oral ibuprofen (10 mg/kg 3 times daily) mixed with Nutella from postoperative day 1 until euthanasia. All animals in the study were postoperatively monitored for clinical signs of pain in accordance with the Danish Animal Experiments Inspectorate’s scoring system for mice and rats.^23^ Euthanasia was performed according to the designated sacrifice schedule listed above by a lethal intraperitoneal injection of Pentobarbital Sodium (100 mg/kg 1 mL/kg).

### Pathological examination

On the day of euthanasia, we removed the chest cage of each animal en-bloc after dissection of the skin, underlying muscles, and connective tissue (**Figure 1**). Lung re-expansion was secured by flushing formaldehyde directly into the trachea after which the thoracic cavity was then systematically examined by a consultant in pathology for pleural adhesions that were graded macroscopically using a modification of a previously described scheme described by Hurewitz et al^24^: 0 = normal pleura, 1 = multifocal adhesions, 2 = diffuse adhesions, 3 = complete obliteration. The pathologist was blinded to which group the animal was assigned. All specimens were fixated in formaldehyde solution for 5 days after which they were re-numbered to ensure continued blinding during pathological microscopic examinations.

**Figure 2.**
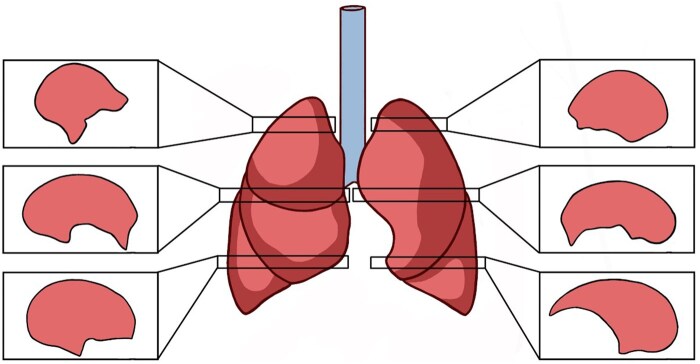
Standard Axial Tissue Section Obtained Bilaterally at the Hilar Level as well as Additional Axial Sections Taken from the Upper and Lower Lobes Bilaterally

For microscopic evaluation, we obtained a standard axial tissue section bilaterally at level of the lung hilum including pleura, lung parenchyma, and the thoracic wall (**Figure 2**). We took additional axial sections from the upper and lower lobes including pleura, lung parenchyma, and the thoracic wall bilaterally (**Figure 2**). All tissue samples were processed through a graded alcohol-xylene series, embedded in paraffin blocks, and stained with hematoxylin and eosin (HE) and Masson’s trichrome.

Particular attention was given to the parameters of tissue collagen deposition, formation of pleural fibrosis, and inflammation. The slides were graded for inflammation and fibrosis and scored using the method described by Hurewitz et al^24^: 0 = absence of inflammation or fibrosis (0%), 1 = mild inflammation or fibrosis (1%-5%), 2 = moderate inflammation or fibrosis (6%-30%), 3 = severe inflammation or fibrosis (> 30%)

### Statistical analysis

Statistical analyses were performed using STATA version 17.0 (StataCorp LLC, Texas, USA). Gross macroscopic and microscopic histopathological grading scores were expressed as mean ± standard deviation (SD). Changes over time within each treatment group were analysed using a Kruskal-Wallis test, followed by Dunn’s test with Bonferroni correction for multiple comparisons when statistically significant differences were detected. Pairwise comparisons between treatment conditions at specific time points were performed using a Wilcoxon signed-rank test for paired data (same animal), and a Wilcoxon rank-sum test for unpaired data (2 different animals). A *P* value < .05 was considered statistically significant.

## RESULTS

Four animals died within 12 hours postoperatively due to hypoxia. These animals were among the first operated on at the beginning of the study, and we assumed that they tolerated bilateral pleural intervention poorly. Consequently, all subsequent animals received supplemental high-flow oxygen postoperatively until the effects of anaesthesia had fully subsided. The 4 animals that died early in the experiment were all replaced with new subjects to maintain the study design.

### Macroscopic findings

Macroscopic adhesion scores remained consistently low across all evaluated time points, with no statistically significant differences observed between autologous blood and talc (**Table 1**). A borderline significant temporal trend was observed within the autologous blood group (Kruskal Wallis *P* = .052), whereas no significant changes over time were detected in the talc group (*P* = .228). The addition of ibuprofen to either treatment modality did not result in significant differences in macroscopic adhesion scores between autologous blood + ibuprofen and talc + ibuprofen. Comparison of autologous blood + ibuprofen to autologous blood alone revealed numerically higher adhesion scores in the combination group but this difference did not reach statistical significance (**Table 1**). Similarly, we did not observe significant differences between talc + ibuprofen and talc alone.

**Table 1.** Macroscopic Mean Grading and Standard Deviation Between and Across Treatment Modalities with the Corresponding *P* Value (italic values)

	Macroscopic adhesions		
Blood patch vs talc	Blood patch	Talc	*Signed rank P value*
Day 2	0.50 (0.41)	0.50 (0.58)	*1.000*
Day 4	0.00 (0.00)	0.00 (0.00)	*1.000*
Day 6	0.25 (0.50)	0.75 (0.65)	*.500*
Day 15	0.00 (0.00)	0.25 (0.50)	*1.000*
Day 30	0.00 (0.00)	0.62 (0.63)	*.250*
*Kruskal-Wallis P value*	*.052*	*.228*	
Blood patch + ibuprofen vs talc + ibuprofen	Blood patch + ibuprofen	Talc + ibuprofen	*Signed rank P value*
Day 15	0.37 (0.48)	0.25 (0.29)	*1.000*
Day 30	0.37 (0.75)	0.12 (0.25)	*1.000*
Blood patch + ibuprofen vs blood patch	Blood patch + ibuprofen	Blood patch	*Rank sum P value*
Day 15	0.37 (0.48)	0.00 (0.00)	*.428*
Day 30	0.37 (0.75)	0.00 (0.00)	*1.000*
Talc + ibuprofen vs talc	Talc + ibuprofen	Talc	*Rank sum P value*
Day 15	0.25 (0.29)	0.25 (0.50)	*1.000*
Day 30	0.12 (0.25)	0.62 (0.63)	*.371*

### Histopathological finding

#### Blood patch vs talc

**Figure 3.**
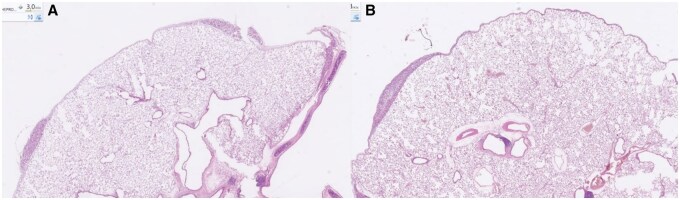
Blood patch (A) vs talc (B) day 15: both specimens show xanthogranulomatous inflammation characterized by cholesterol crystals, multinucleated giant cells, and lymphocytic infiltration. Early pleural fibrosis is observed, presented as loose and edematous connective tissue with scattered fibroblasts. After blood patch (A), the fibrosis appears more compact, and in both specimens corresponds to grade 3

We found no statistically significant differences between autologous blood and talc pleurodesis regarding inflammation or fibrosis at any individual time point (**Figure 3**). Inflammation peaked at day 6 after blood patch (mean 2.62) and at day 30 after talc (mean 2.37), while fibrosis increased progressively in both groups (**Table 2**). Kruskal-Wallis analysis showed significant time-dependent variation for both inflammation and fibrosis in each group (inflammation: *P* = .005 and *P* = .019, fibrosis: *P* < .001) and post hoc analysis identified significantly higher inflammation at day 6 vs days 2 and 4 after blood patch (*P* < .001), and at day 30 compared to days 2 and 4 after talc (*P* = .049 and *P* = .015).

**Table 2.** Microscopic Mean Grading and Standard Deviation Between and Across Treatment Modalities with the Corresponding *P* Value (italic values)

	Inflammation			Fibrosis		
Blood patch vs talc	Blood patch	Talc	*Signed rank P value*	Blood patch	Talc	*Signed rank P value*
Day 2	1.25 (0.71)	1.00 (0.76)	*.687*	0.50 (0.53)	0.37 (0.74)	*.687*
Day 4	1.12 (1.13)	0.75 (0.89)	*.500*	1.50 (1.07)	1.25 (1.04)	*.500*
Day 6	2.62 (0.52)	1.87 (1.25)	*.250*	2.25 (1.04)	1.62 (0.92)	*.218*
Day 15	1.87 (0.83)	1.62 (1.06)	*.750*	2.37 (0.52)	2.50 (0.76)	*1.000*
Day 30	2.12 (0.64)	2.37 (1.06)	*.656*	2.75 (0.46)	3.00 (0.00)	*.500*
*Kruskal-Wallis P value*	*.005*	*.019*		*<.001*	*<.001*	

	Inflammation			Fibrosis		
Blood patch + ibuprofen vs talc + ibuprofen	Blood patch + ibuprofen	Talc + ibuprofen	*Signed rank P value*	Blood patch + ibuprofen	Talc + ibuprofen	*Signed rank P value*
Day 15	1.37 (0.92)	1.25 (0.87)	*1.000*	1.37 (1.06)	1.12 (0.99)	*.625*
Day 30	1.62 (1.06)	1.75 (1.16)	*1.000*	1.75 (0.89)	2.00 (1.31)	*.625*

	Inflammation			Fibrosis		
Blood patch + ibuprofen vs blood patch	Blood patch + ibuprofen	Blood patch	*Rank sum P value*	Blood patch + ibuprofen	Blood patch	*Rank sum P value*
Day 15	1.37 (0.92)	1.87 (0.83)	*.364*	1.37 (1.06)	2.37 (0.52)	*.063*
Day 30	1.62 (1.06)	2.12 (0.64)	*.352*	1.75 (0.89)	2.75 (0.46)	*.024*

	Inflammation			Fibrosis		
Talc + ibuprofen vs talc	Talc + ibuprofen	Talc	*Rank sum P value*	Talc + ibuprofen	Talc	*Rank sum P value*
Day 15	1.25 (0.87)	1.62 (1.06)	*.425*	1.12 (0.99)	2.50 (0.76)	*.008*
Day 30	1.75 (1.16)	2.37 (1.06)	*.252*	2.00 (1.31)	3.00 (0.00)	*.076*

#### Blood patch + ibuprofen vs talc + ibuprofen

When we administered ibuprofen postoperatively, inflammation and fibrosis grading remained lower across both groups although not statistically significant (**Table 2**). At day 15 and 30, inflammation scores were comparable between the blood patch + ibuprofen and talc + ibuprofen groups (*P* = 1.000). Similarly, fibrosis scores did not differ significantly (day 15: *P* = .625; day 30: *P* = .625).

#### Blood patch vs blood patch + ibuprofen

**Figure 4.**
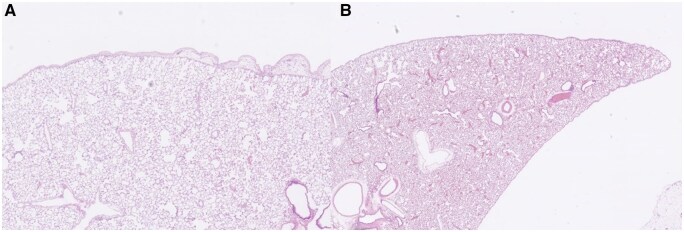
Blood patch (A) vs blood patch + ibuprofen (B) day 30: after blood patch alone (A), early fibrotic changes are observed, with an extent corresponding to grade 3, while fibrosis is less pronounced in animals treated with ibuprofen postoperatively (B)

Ibuprofen appeared to reduce both inflammation and fibrosis after autologous blood pleurodesis (**Table 2**, **Figure 4**). Inflammation was lower at both day 15 and day 30, but these differences were not statistically significant. Fibrosis scores, however, showed a significant reduction at day 30 in the ibuprofen group (1.75 vs 2.75, *P* = .024) (**Figure 4**), and a near-significant reduction at day 15 (1.37 vs 2.37, *P* = .063).

#### Talc vs talc + ibuprofen

**Figure 5.**
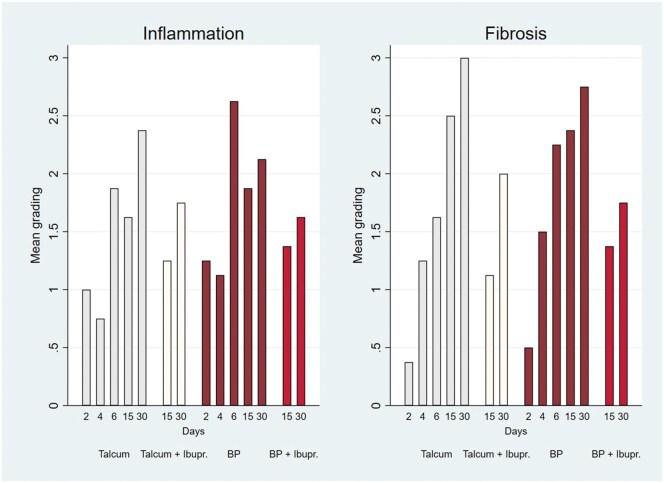
Mean Grading of All 4 Treatment Modalities with Respect to Inflammation and Fibrosis Over Time. Abbreviations: BP: blood patch; Ibupr.: ibuprofen

Compared to talc alone, the addition of ibuprofen was associated with a reduction in both inflammation and fibrosis (**Table 2**, **Figure 5**). Inflammation scores were lower with ibuprofen but not statistically significant (day 15: 1.25 vs 1.62, *P* = .425; day 30: 1.75 vs 2.37, *P* = .252). Fibrosis was significantly lower in the talc + ibuprofen group at day 15 (1.12 vs 2.50, *P* < .008), while the difference at day 30 approached significance (2.00 vs 3.00, *P* = .076).

#### Comment

For 2 decades management of persistent air leakage at our institution include the option to perform autologous blood pleurodesis by intrapleural instillation of 120 mL of autologous venous blood but the decision was left to the surgeons. In some patients, the air leakage appears to cease—in others not—and the exact mechanism is not known. In the present study, we aimed to investigate this clinical practice as closely as possible in an experimental setting by characterizing the histopathological changes over time following autologous blood using talc as a positive control. We also aimed to investigate possible changes by postoperative administration of ibuprofen. Our results did not demonstrate any significant difference in pleurodesis efficacy between autologous blood and talc when assessed by macroscopic adhesion scores or by histopathological grading of inflammation and fibrosis. We did however observe time-dependent variation in inflammation and fibrosis response in both groups and noticed peak inflammation earlier in the autologous blood group (day 6) compared to talc (day 30), suggesting a temporal difference in pleural reaction dynamics.

Our findings are consistent with those reported by Kapicibasi et al,^4^ who also did not find any significant differences between autologous blood and talc in pleurodesis efficacy. Results from both studies, however, contrast with the findings by Mitchem et al,^2^ who reported a higher degree of inflammation and fibrosis following talc instillation compared to autologous blood in a rabbit model. One limitation of the previous studies is that pleurodesis scoring was performed only at a single time point (day 30), leaving uncertainty about possible changes and the underlying mechanism responsible for the clinical effect of blood patch pleurodesis in the immediate postoperative period. As previously suggested,^2^^,^^3^ it remains unclear whether the early clinical benefit of autologous blood is primarily due to an inflammatory/fibrotic reaction or to a mechanical sealing effect via clot formation sealing the bronchopulmonary fistula.

The lack of statistically significant difference between autologous blood and talc pleurodesis in terms of overall inflammatory or fibrotic response in our study suggests that both agents induce a comparable pleural reaction. This aligns with clinical observations of similar efficacy in managing persistent air leakage,^10^^,^^11^^,^  ^13^^,^  ^18^ although the underlying mechanisms may differ. The earlier peak in inflammation following autologous blood pleurodesis may reflect a more rapid but potentially shorter-lived response, possibly related to the mechanical “plug” effect combined with moderate biological activity.

In our study, the addition of ibuprofen reduced both inflammation and fibrosis with autologous blood and talc instillation, but the differences varied over time and did not reach statistical significance at all time points. There was a consistent trend towards lower scores in the NSAID-treated animals indicating a potential inhibitory effect of ibuprofen on pleurodesis formation consistent with findings in the medical literature. Thus, a recent clinical study published in *Nature* demonstrated that NSAIDs attenuate pleural adhesion formation,^22^ and previous experimental studies showed reduced pleural adhesion formation after talc instillation when corticosteroids or NSAIDs were administered concomitantly.^25^^,^^26^ A recent animal study investigated the impact of autologous blood pleurodesis in rats treated postintervention with diclofenac or paracetamol. The authors reported that both agents significantly reduced the degree of inflammation and fibrosis.^5^ In that study, however, histopathological evaluation was limited to sections taken from macroscopically visible adhesions without standard sampling across pleural surfaces. Furthermore, the animals were treated with intraperitoneal injections of diclofenac and paracetamol for 5 consecutive days, which does not reflect clinical practice.

We used talc pleurodesis as positive control in the contralateral hemithorax because talc has long been recognized as one of the most effective sclerosing agents for inducing pleurodesis and is widely used both experimentally and clinically. Its efficacy in producing robust pleural inflammation, fibrosis, and durable adhesion formation has been demonstrated consistently in various animal models^2^^,^  ^4^ as well as in clinical studies.^8^^,^  ^19^ By instilling talc in the contralateral hemithorax, we were able to establish a reliable intra-animal benchmark to compare pleurodesis response from autologous blood patch. In addition, the paired design minimized biological variability and ensured that each animal served as its own control, thereby enhancing sensitivity to detect subtle differences in histopathological response over time. Assessing the anti-inflammatory impact of ibuprofen on both autologous blood and talc-induced pleurodesis in a shared physiological environment reduced inter-animal variability and permitted direct within-subject comparisons of drug effect. This enhanced the reliability of grading inflammatory and fibrotic responses, and provided a unique opportunity to determine whether NSAID-mediated modulation differs between both pleurodesis mechanisms.

#### Limitations

This study has several limitations. First, we did not include a true negative control group with no intrapleural intervention. While the use of talc as a positive control allowed for meaningful intra-animal comparison, the absence of a negative control group limits our ability to determine the baseline histopathological response to procedural intervention alone. Nevertheless, the main finding in our experimental study remains interesting that we found no significant differences between autologous blood and talc regarding macroscopic adhesion scores or grading of inflammation and fibrosis. Second, the overall sample size was relatively small, particularly in later subgroups where animals were further stratified based on ibuprofen treatment. Although statistical comparisons were performed using appropriate non-parametric methods, the study may have been underpowered to detect smaller but potentially clinically relevant differences between the groups.

## AUTHOR CONTRIBUTIONS

All co-authors have contributed significantly to the conception, design, data collection, analysis, and interpretation of the work presented in the study. Each co-author has reviewed and approved the final version of the manuscript.

## Data Availability

The data underlying this article will be shared on reasonable request to the corresponding author.
